# Epidemiology, Diagnosis and Treatment of Acute Promyelocytic Leukemia in Children: the Experience in China

**DOI:** 10.4084/MJHID.2012.012

**Published:** 2012-03-10

**Authors:** Li Zhang, Xiaofan Zhu

**Affiliations:** Department of Pediatrics, Institute of Hematology and Blood Diseases Hospital, Chinese Academy of Medical Sciences and Peking Union Medical College, Tianjin, PR China

## Abstract

Acute promyelocytic leukemia (APL) is the subtype of acute myeloid leukemia characterized by an accumulation of abnormal promyelocytes in bone marrow, a severe bleeding tendency and the presence of the chromosomal translocation t(15;17) or variants. APL, the most fatal type of leukemia two decades ago, is highly curable with current treatment strategies. There is evidence that the incidence of APL varies across ethnic groups and that genetic factors play a role in the etiology of APL. And there are some difference between children and adults in APL.^1^–^3^ The limited data of children available in many developing countries suggest that the rate of early mortality is high and that long-term survival is poor. Death from bleeding and infection during chemotherapy, relapse and treatment abandonment are among the main cause of treatment failure in APL children as well in adults.^2^ The status of children APL treatment in China has not been described in general.

Here we describe the epidemiology and treatment of APL in children in China. In addition, we review the results of a survey of its clinical manifestations and outcome in China.

## Epidemiology of APL

### Demographics

The real incidence of APL is not known. The lack of population-based registries in China made it difficult to determine the real incidence of APL. The incidence of APL was estimated on the basis of its relative frequency among other AML subtypes in large clinical trials. The Hematology and Blood Diseases Hospital of Chinese Academy of Medical Sciences and Peking Union Medical College has provided information about child APL.^4^ In this registry, 51(31.6%) of the 141 cases of AML registered between 1996 and 2004. In the Children’s hospital of Zhejiang University School of Medicine registry, 49(26.5%) of the 185 cases of AML registered between 1997 and 2005.^5^ The percentage of APL could be higher in children than in adults.^6^

These data is somewhat higher than the 5% to 13% reported by many large clinical trials and single institutions in the US.^7^ Also of note, this incidence is similar to Latin American background patients.^7^ The difference of ethnicity may be the explanation. In addition, admission bias have to be considered for the favorable outcome in APL. Population-based cancer registries is needed to confirm the real incidence of APL.

### Other factors

Most of people in China is Han descent. So there is no data about epidemiology feature of APL in different ethnic groups.

## Diagnosis and Treatment of Acute Promyelocytic Leukemia

### Diagnosis of APL

The French-American-British (FAB) classification recognizes two main morphologic APL subtypes, including a more frequent hypergranular form (M3) featured by abnormal dysplastic promyelocytes with abundant cytoplasmic granules and Auer bodies, and a less frequent microgranular form characterized by abnormal promyelocytes with minimal cytoplasmic granulation, reniform or bilobed nuclei, and can be confused morphologically with monocytic subtypes of AML.^3^ There is no data about the rate of hypergranular form and microgranular form in the studies in china. Zhang et al described one patients who was diagnosed as acute monocytic leukemia initially died at 4 days after treated with cytarabine.^8^

APL immunophenotype is characterized by frequent expression of CD13 and CD33and rare expression of HLA-DR, CD34, CD10, CD7, CD11b, and CD14.^3^

At the genetic level, APL is for the most characterized by a unique reciprocal chromosome translocation t(15;17)(q22;q11–12), leading to a fusion between the promyelocytic (PML) gene on chromosome 15 and the retinoic acid receptor-α (RARa) gene on chromosome 17. In our study^8^ including 65 children cytogenetics was performed in 47 patients, 20 (42%) patients had the t(15;17)(q22;q11–12), 10 (21%) had additional cytogenetic abnormalities to t(15;17)(q22;q11–12), 14 (30%) had a normal pattern, in 3 other cytogenetic abnormalities were present.

The additive chromosomal changes was not associated with a different outcome.

Most of these patients was diagnosed by the presence of the fusion protein PML/RARa detected by polymerase chain reaction. In China, minimal residual disease (MRD) monitoring based on the detection of PML/RARa transcripts was mainly performed utilizing the reverse transcription polymerase chain reaction (RT-PCR). Some of the hospitals can monitor MRD by real-time quantitative PCR (RQ-PCR). In our study, the rate of bcr1, bcr2 and bcr3 PML/RARa subtype mRNA is 71.8%, 7.7% and 20.5%, respectively. And we analyzed the prognostic value of the standardized PML/RARa RQ-PCR assay in children with newly diagnosed APL children. At the end of the third consolidation course, no patient was RQ-PCR-positive except for one who relapsed. Our results indicated that patients with a negative RQ-PCR result after induction were correlated with a lower probability of relapse.^9^

### Treatment of APL

Optimal management of APL requires prompt diagnosis and immediate initiation of treatment. Before the late 1980s, APL was treated the same as other AML subtypes. Although in the developed country, the remission rate was much lower than 70%.^10^,^11^ The discovery of the clinical efficacy of all-trans retinoic acid (ATRA) by Chinese hematologists turned a new page in the history of leukemia therapy.^12^ In the mid-1990s, the first controlled clinical trial of arsenic trioxide (ATO) further improved the clinical outcome of refractory or relapsed APL.^13^

Table 1 lists selected clinical, laboratory, and outcome data from children with APL obtained from China which had been published recently.^5^,^7^,^14^–^18^ Cytogenetic and molecular diagnosis of APL was confirmed in most of the centers. Conventional RT-PCR had been widely used for therapeutic monitoring of APL. ATRA was used in the management of most of these centers, but in harbin, 19 patients received ATO without ATRA. The median age was around 9 years. There was a predominance of boys (M:F ratio, 1.06–2.5). The median white blood cell (WBC) count at diagnosis varied from 3.46×10^9^/L to 10.5×10^9^/L, and the median platelet count from 21×10^9^/L to 36.5×10^9^/L. Rate of complete remission (CR) ranged from 85.7% in Shen yang to 95.1% in Zhe jiang. Abandonment or refusal of treatment was documented in 21 cases. Also of note, the abandonment rate decreased from 31.25% (5/16) to none after 2005 in Guang zhou.^15^ The improvement may be due to the better economic status and improved financial support recently. Except for the study with high rate of abandonment in Guang zhou, most of the other studies suggested the similar outcomes when compared with the other large series of children APL groups treated with ATRA and chemotherapy.^19^ The outcome of children with APL in China comparatively better in developing countries.^2^ Treatment with ATRA as soon as possible after morphologic diagnosis in the bone marrow, with experience in the supportive care and management of complication, and added ATO in the salvage treatment may be the explanation.

As described in table 1, the relapse rate is difference among different studies.^5^,^7^,^13^–^17^ The patients who relapsed will be treated with ATO±ATRA. Occasionally, the relapsed patients will give up treatment.

### Special treatment issues for Children with APL

A special note is necessary for the risk of cardiomyopathy, a real threat for children with APL treated with regimens that utilize high doses of anthracyclines. Van Dalen et al suggest the risk of developing clinical heart failure was dose-dependent, increasing from 0% for 150 mg/m^2^ of anthracyclines up to 14.3% for 600 mg/m^2^.^20^ In our experience there was one 5-year-old boy who developed severe congestive heart failure when the total dose of anthracycline was reach to 530 mg/m^2^ body surface due to molecular relapse.^21^ However, no long-term data about cardiotoxicity of anthracycline can be described in China so far.

Another issue of special importance in children with APL is the difficulty in swallowing the capsule of ATRA. A novel intravenous liposomal formulation of ATRA has been shown to be effective in inducing CR.^22^ However, this form is not commercially available yet. Then ATO may be considered for these patients.

#### Current Situation in China

Several years ago, financial support from the government was lack. Most families did not have medical insurance. So they could not afford the costly medical care. Recently, financial support from the society (such as China Red Cross foundation: www.crcf.org.cn/xts/index.asp) and the government (www.gov.cn/zwgk/2010-06/10/content_1624580.htm) had increased the coverage of medical insurance to ensure that most families can afford the treatment costs. Since 2009, more than 3,000 children with leukemia received the financial support from the China Red Cross foundation. However, there are still more patients needs financial support so as to be treated regularly. In addition, setting up the population-based cancer registries in China is necessary to obtain data with sufficient quality for international comparison.

Although the outcome of children with APL in China is improved, we hope to establish a relationship with hematologists/oncologists in the world to promote international communication.

## Figures and Tables

**Table 1:** Selected clinical, laboratory, and outcome features of children with acute promyelocytic leukemia (APL) in China.

Authors (et al)	Li Zhang^8^	Jing Chen^14^	Xue-Qun Luo^15^		Xiao-Jun Xu^5^	Jin Zhou^16^	Hong Wang^17^	Li Zhang^18^
City	Tianjin	Shanghai	Guangzhou		Zhejiang	Harbin	Shenyang	Tianjin
Year	2008	2008	2009		2010	2010	2010	2011
Period(Year/Month)	1999/1–2005/12	1998/7–2006/5	1999/12–2004/12	2005/1–2008/1	1997/1–2005/12	2001/8–2007/1	2000/1–2009/1	1999/1–2003/12
Diagnosis methods Chromosome/RT-PCR/other	47/50/6	21/21/14	NA	NA	NA	11/16/0	10/18/7	7/30/0
No, patients	65	35	16‡	14	41§	19¶	35	37
Age,median(range)	13 (2–18)	9.3 (0.4–15)	9 (0.5–15)		9.9 (1.5–13.8)	4–15	NA	2–14
Sex,male/femal (ratio)	36/26 (1.38)	21/14(1.5)	10/6 (1.66)	10/4 (2.5)	22/19 (1.15)	11/8 (1.38)	22/13 (1.69)	19/18 (1.06)
WBC×10^9^/L,median(range)	3.46 (0.9–580)	10.5 (0.9–47.8)	8 (1–143)	7 (2–187)	5.2 (2.0–221.8)	4.8 (1.0–89.6)	NA	1.12–580
WBC≥10×10^9^/L(%)	17(26.2)	11(33.3)	NA	NA	NA	7	NA	10
WBC<10×10^9^/L (%)	48(73.8)	22(66.6)	NA	NA	NA	12	NA	27
PLT×10^9^/L,median (range)	23.0 (4–153)	NA	21 (8–267)	26 (9–128)	35 (1.5–216)	36.5 (10–102)	NA	6–153
PLT≥40×10^9^/L	19(29.2)		NA	NA	NA	8	NA	NA
PLT<40×10^9^/L	46(70.8)		NA	NA	NA	11	NA	NA
Induction treatment	ATRA±ATO	ATRA+CT	ATRA+CT	ATRA+CT	ATRA	ATO	ATO±ATRA	ATRA±ATO
CR (No.) (%)	59(90.8)	33(94.3)	9(90)	13(92.9)	39(95.1)	17(89.5)	30(85.7)	35(94.6)
Early death (No.)(%)	6(9.2)*	2(5.7)	1(10)	1(7.1)	2(4.9)	2(10.5)	3(8.6)	2(5.4)
Other							2(5.7)**	
Consolidation treatment	CT	CT†	CT†	CT	CT†	ATO	CT	CT
Follow-up (months)	38(1–93)	49(4–103)			69(28–135)	53(23–76)	10–108	76(2–129)
Event-free survival (%)	77.5% (5–year)	73.4% (7–year)	37.5% (3.5year)	79.6% (3.5year)	63.5% (7–year)	72.7% (5–year)	78.3% (5–year)	79.2% (5–year)
Disease-free survival(%)	85.4% (5–year)	73.4% (7–year)						83.7% (5–year)
Overall survival (%)	88.9% (5–year)	91.2% (7–year)			66.9% (7–year)	83.9% (5–year)	82.7% (5–year)	91.5% (5–year)
